# Object Tracking Using Local Multiple Features and a Posterior Probability Measure

**DOI:** 10.3390/s17040739

**Published:** 2017-03-31

**Authors:** Wenhua Guo, Zuren Feng, Xiaodong Ren

**Affiliations:** Systems Engineering Institute, State Key Laboratory for Manufacturing Systems Engineering, Xi’an Jiaotong University, Xi’an 710049, China; fzr9910@mail.xjtu.edu.cn (Z.F.); rxd@mail.xjtu.edu.cn (X.R.)

**Keywords:** object tracking, multiple features, posterior probability measure, centroid iteration

## Abstract

Object tracking has remained a challenging problem in recent years. Most of the trackers can not work well, especially when dealing with problems such as similarly colored backgrounds, object occlusions, low illumination, or sudden illumination changes in real scenes. A centroid iteration algorithm using multiple features and a posterior probability criterion is presented to solve these problems. The model representation of the object and the similarity measure are two key factors that greatly influence the performance of the tracker. Firstly, this paper propose using a local texture feature which is a generalization of the local binary pattern (LBP) descriptor, which we call the double center-symmetric local binary pattern (DCS-LBP). This feature shows great discrimination between similar regions and high robustness to noise. By analyzing DCS-LBP patterns, a simplified DCS-LBP is used to improve the object texture model called the SDCS-LBP. The SDCS-LBP is able to describe the primitive structural information of the local image such as edges and corners. Then, the SDCS-LBP and the color are combined to generate the multiple features as the target model. Secondly, a posterior probability measure is introduced to reduce the rate of matching mistakes. Three strategies of target model update are employed. Experimental results show that our proposed algorithm is effective in improving tracking performance in complicated real scenarios compared with some state-of-the-art methods.

## 1. Introduction

Among the numerous subjects in computer vision, object tracking is one of the most important fields. It has many applications such as human computer interaction, video analysis, and robot control systems.

Many object tracking algorithms have been brought up in the last decades. Welch [1] proposed a Kalman filter-based algorithm considering Gaussian and linear problems to track one’s pose in interactive computer graphics. Later, a particle filter-based approach was introduced with respect to non-Gaussian and non-linear systems [2,3]. Other common trackers used include optical flow-based tracking [4], multiple hypothesis tracking [5,6], and kernel-based tracking [7,8]. Recently, João F. Henriques et al. [9] proposed a new kernel tracking algorithm called high-speed tracking with kernelized correlation filters that have been widely used. Unlike other kernel algorithms, the method has the exact same complexity as its linear counterpart.

Though these algorithms have been successful in many real scenes, they are still confronted with challenging problems, such as illumination changes, object occlusions, image noises, low illumination, fast motions and similarly colored backgrounds. One of the effective solutions is the mean-shift algorithm which can handle object partial occlusions and background clutters [10,11,12]. Mean-shift is a non-parametric pattern matching tracking algorithm. It uses the color histogram as the target model and the Bhattacharyya coefficient as the similarity measure. The location of the target is obtained by an iterative procedure [10]. The performance of the algorithm is determined by the similarity measure and the target feature. Because of the background interference, the tracking result may easily get biased or be completely wrong. The location of the target obtained by the Bhattacharyya coefficient [7] or other similarity measures, such as normalized cross correlation, histogram intersection distance [13], and Kullback–Leibler divergence [14] may not be the ground truth. To improve the accuracy of object matching, a maximum posterior probability measure was proposed [15]. It takes use of the statistical feature of the searching region and can effectively reduce the influence of background and emphasize the importance of the target.

In some scenes with dramatic intensity or color changes, the effectiveness of the color decreases. Thus, it is desirable that some additional features should be used as a complement to color to improve the performance of the tracking system [16,17]. For example, Collins et al. [18] presented an online feature selection algorithm based on a basic mean-shift approach. The method can adaptively select the best features for tracking. They only used the RGB histogram in the algorithm, but it can be extended to other features. Wang et al. [19] proposed integrating color and shape-texture features for reliable tracking, and their method was also based on the mean-shift algorithm. Ning et al. [20] presented a mean-shift algorithm using the joint color-texture histogram, which proved to be more robust and insensitive than the color. Most of these methods used multiple features to describe the target model in order to reduce the mistakes of tracking systems. Unfortunately, color, shape-texture silhouettes or other traditional features can not track the target in some special scenes with variably scaled images or rotated images. In recent years, some new features have been proposed to solve these problems including Scale Invariant Feature Transform (SIFT) [21], Principal Components Analysis-Scale Invariant Feature Transform (PCA-SIFT) [22], Gradient Location and Orientation Histogram (GLOH) [23], Speed-up Robust Feature(SURF) [24], and Fast Retina Keypoint (FREAK) [25], just to name a few. Among them, a texture feature named the local binary pattern (LBP) [26] has been widely used in computer vision [27] due to its advantages of fast computation and rotation invariance. Recently, some improvements have been made based on the LBP such as the center-symmetric local binary pattern (CS-LBP) [28] and the local ternary pattern (LTP) [29].

This paper proposes a centroid iteration algorithm with multiple features based on a posterior probability measure [15] for object tracking. The main goal is to solve the difficulties in real scenes such as similarly colored backgrounds, object occlusions, low illumination color image and sudden illumination changes. The proposed algorithm consists of a target model construction step and a localization step. We improve the LBP descriptor to the DCS-LBP descriptor. For further improvement, a simplified version of the DCS-LBP is used, which we call the SDCS-LBP. It can describe important information of the image (the edge, the corner and so on). Then, this new texture feature and the color are combined to constitute the multiple features used in the target model, which we call the color and texture (CT) feature in this paper. After obtaining the target, three strategies for updating the target model are presented to reduce the tracking mistakes.

The rest of the paper is organized as follows: in Section 2, a local color texture feature based on the DCS-LBP along with its simplified form is introduced. In Section 3, the proposed tracking algorithm is illustrated in detail. Experimental results are shown in Section 4. Section 5 draws conclusions.

## 2. Multiple Features

Feature descriptors are very important in matching-based tracking algorithms, especially for applications in real scenes. In some simple scenes, color can work well because it distinguishes the targets from the background easily and contains a lot of useful information of the target. However, in complex scenes containing similarly colored backgrounds, object occlusions, low illumination color image and sudden illumination changes, the tracker only using the color feature may easily miss the target. One of the solutions is to integrate multiple features in the target model for reliable tracking.

### 2.1. Local Binary Patterns (LBPs)

**Figure 1:**
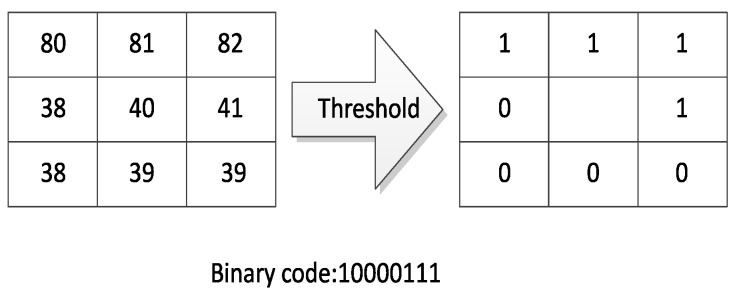
The original LBP code.

The LBP is an illumination invariant texture feature. The operator uses the gray levels of the neighboring pixels to describe the central pixel. The texture model $LBP_{P,R}$ is expressed as follows [26]:

(1) $$\begin{array}{c} LBP_{P,R}=\sum_{i=0}^{P-1}s\left(g_{i}-g_{c}\right)2^{i},\\ s\left(x\right)=\left\{\begin{array}{cc} 1, & x\geq 0,\\ 0, & x<0, \end{array}\right. \end{array}$$

where *P* is the number of the neighbours and *R* is the radius of the central pixel. $g_{c}$ denotes the gray value of the central pixel and $g_{i}$ denotes that of the *P* neighbours with i=0,...,P−1, and s(x) represents the sign function. Figure 1 gives an example of the LBP code when P=8 and R=1.

There are two extensions of the LBP [26]. The first one is to make the LBP as a rotation invariant feature as proposed by Ojala et al. [26]. It is defined as:

(2) $$LBP_{P,R}^{ri}=min\left(ROR\left(LBP_{P,R},i\right)|i=0,1,\cdots ,P-1\right),$$

where ROR(x,i) performs a circular bit-wise right shift on the P_bit number *x* by *i* times. Equation (2) selects the minimal number to simply the function. They explained that there were 36 rotation invariant LBP codes at P=8, R=1. The second one is the uniform LBP, which contains at most one 0–1 and one 1–0 transition when viewed as a circular bit string. The uniform LBP codes contain a lot of useful structural information. Ojala et al. [26] observed that although only 58 of the 256 8-bit patterns were uniform, nearly 90% of all observed image neighborhoods were uniform and many of the remaining ones contained noise. The following operator $LBP_{8,1}^{riu2}$ is a uniform and rotation invariant pattern with Uvalue of at most 2:

(3) $$\begin{array}{c} LBP_{P,R}^{riu2}=\left\{\begin{array}{cc} \sum_{i=0}^{P-1}s\left(g_{i}-g_{c}\right)2^{i}, & U(LBP_{P,R}\leq 2),\\ P+1, & \mathrm{otherwise}, \end{array}\right.\\ U\left(LBP_{P,R}\right)=|s\left(g_{P-1}-g_{c}\right)-s\left(g_{0}-g_{c}\right)|+\sum_{i=1}^{P-1}\left|s\left(g_{i}-g_{c}\right)-s\left(g_{i-1}-g_{c}\right)\right|. \end{array}$$

**Figure 2:**
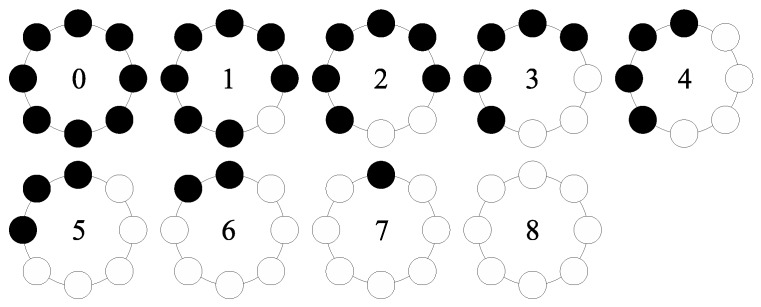
Nine uniform patterns of $LBP_{8,1}^{riu2}$.

If we set P=8, R=1, the nine most frequent patterns with index from 0 to 8 are selected from the 36 different patterns, which are the rotation invariant patterns as shown in Figure 2.

### 2.2. Center-Symmetric Local Binary Patterns (CS-LBPs) and Local Ternary Patterns (LTPs)

In Section 2.1, it can be seen that LBP codes have a long histogram, which require lots of calculations. Heikkilä et al. [28] designed a method by comparing the neighboring pixels in order to reduce computation. They calculated the center-symmetric pairs of the pixels as defined in the following function:

(4) $$\begin{array}{c} C\mathrm{S-L}BP_{P,R}=\sum_{i=0}^{\frac{P}{2}-1}s\left(g_{i}-g_{i+\frac{P}{2}}\right)2^{i},\\ s\left(x\right)=\left\{\begin{array}{cc} 1, & x\geq T,\\ 0, & \mathrm{otherwise}. \end{array}\right. \end{array}$$

This operator halves the calculations of LBP codes at the same neighbors. The LBP threshold depends on the central pixel, which makes the LBP sensitive to noise especially in flat regions of the image while the CS-LBP threshold is a constant value *T* that can be adjusted.

Tan et al. [29] extended the LBP to 3-valued codes, called the local ternary pattern (LTP). They set the codes around $g_{c}$ in a zone of width ±T to one. The codes above it are set to 2 and the ones below it are set to 0. It is defined as:

(5) $$\begin{array}{c} LTP_{P,R}=\sum_{i=0}^{P-1}s\left(g_{i}-g_{c}\right)3^{i},\\ s\left(x\right)=\left\{\begin{array}{cc} 2, & x\geq T,\\ 1, & -T<x<T,\\ 0, & x\leq -T. \end{array}\right. \end{array}$$

Here, *T* is the same threshold as the CS-LBP. Thus, the LTP is more insensitive to noise than the CS-LBP. However, it is no longer invariant to gray-level transformations.

### 2.3. Double Center-Symmetric Local Binary Patterns (DCS-LBPs)

In Section 2.2, it is analyzed that the CS-LBP is more efficient than the LBP in calculation, but they are both sensitive to noise. The LTP is insensitive to noise, but its computation is too complex. A simple way is to combine the LTP and the CS-LBP, which yields the CS-LTP. It is defined as:

(6) $$\begin{array}{c} C\mathrm{S-L}TP_{P,R}=\sum_{i=0}^{\frac{P}{2}-1}s\left(g_{i}-g_{i+\frac{P}{2}}\right)3^{i},\\ s\left(x\right)=\left\{\begin{array}{cc} 2, & x\geq T,\\ 1, & -T<x<T,\\ 0, & x\leq -T. \end{array}\right. \end{array}$$

By definition, the CS-LTP retains the advantages of the CS-LBP and the LTP, but the ternary values are hard to calculate in the image.

Thus this motivates us to generate a DCS-LBP operator. The operator is divided into two parts: $DCS-LBP_{P,R}^{\left(upper\right)}$, in which the gray levels of the center-symmetric pixels above *T* are quantized to one while those below *T* are quantized to zero, and $DCS-LBP_{P,R}^{\left(lower\right)}$, in which the center-symmetric pixels on the other side below −T are quantized to one while those below *T* are quantized to zero.

(7) $$\begin{array}{c} \left\{\begin{array}{cc} DCS-LBP_{P,R}^{upper}=\sum_{i=0}^{\frac{P}{2}-1}s_{1}\left(g_{i}-g_{i+\frac{P}{2}}\right)2^{i}, & s_{1}\left(x\right)=\left\{\begin{array}{cc} 1, & x\geq T,\\ 0, & \mathrm{otherwise}, \end{array}\right.\\ DCS-LBP_{P,R}^{lower}=\sum_{i=0}^{\frac{P}{2}-1}s_{2}\left(g_{i}-g_{i+\frac{P}{2}}\right)2^{i}, & s_{2}\left(x\right)=\left\{\begin{array}{cc} 1, & x\leq -T,\\ 0, & \mathrm{otherwise}. \end{array}\right. \end{array}\right. \end{array}$$

*T* is the threshold used to eliminate the influence of weak noise. The value of *T* determines the anti-noise capability of the operator. The upper-part and the lower-part of the DCS-LBP should be calculated separately and then be combined together for use. By definition, there are $2\times 2^{\frac{P}{2}}$ different values, which are much less than the basic LBP ($2^{P}$) and the LTP ($3^{P}$), and are close to the CS-LBP ($2^{\frac{P}{2}}$) and the CS-LTP ($3^{\frac{P}{2}}$). When P=8, R=1, the DCS-LBP has 32 different values. Table 1 shows examples of all of these five local patterns. The first row are three local parts of an image including texture flat areas, texture flat areas with noise, and texture change areas. The threshold is set to be 5. It can be seen that the LBP and the CS-LBP can not exactly distinguish between texture flat and change areas. The other three patterns are distinguishable and are all insensitive to noise, among which the computational complexity of the DCS-LBP is lower than the other two.

**Figure 3:**
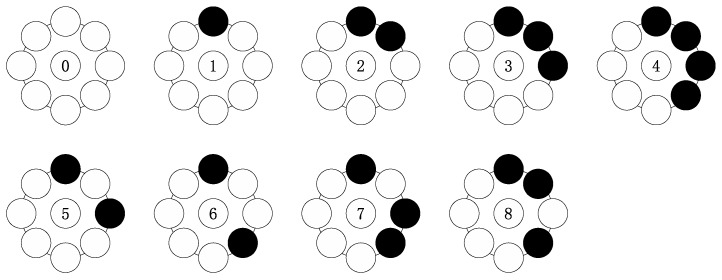
The nine rotation invariant patterns of the DCS-LBP.

It should be noted that there is a great amount of redundant information in the DCS-LBP, which might cause matching errors. Thus, further optimization is necessary. The DCS-LBP patterns also have the rotation invariant identity as shown in Figure 3. There are nine rotation invariant patterns. Similarly, both $DCS-LBP_{P,R}^{\left(upper\right)}$ and $DCS-LBP_{P,R}^{\left(lower\right)}$ have the same uniform patterns as the LBP. Pattern 5 to Pattern 8, which cannot describe the primitive structural information corresponding of the local image, are not uniform patterns. Pattern 0 to Pattern 4 each has its identity. Pattern 0 and Pattern 1 represent noise points, dark points and smooth regions. Pattern 2 represents the terminal. Pattern 3 represents angular points. Pattern 4 represents boundary. Thus, we improve the DCS-LBP to its simplified version (called SDCS-LBP), which retains only the patterns with index from 0 to 4.

### 2.4. Local Color Texture Feature (CT Feature)

Feature representation of the target model is very important for mean-shift based tracking algorithms. The original mean-shift algorithm selects the RGB color space (16×16×16 = 4096) as the features. However, in real scenes which contain similarly colored background, object occlusion, low illumination color image and sudden illumination changes, the original mean-shift algorithm can not track the target continuously. Inspired by [16], we consider designing a new feature combining the color and the texture.

**Figure 4:**
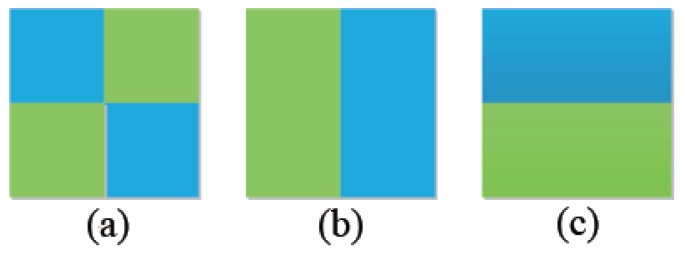
A particular target model.

This paper chooses to use the HSV color space, which contains Hue, Saturation and Value. The Value, which is measured with some white points, is often used for description of surface colors and remains roughly constant even with brightness and color changes under different illuminations. Hence, we replace the Value with the SDCS-LBP in the HSV space as the target model. The new feature which combines the color and the texture is called the CT feature in this paper. The CT feature can be considered as a special texture feature (terminal, angular point, boundary and some special points) with a certain color. The HSV color space is reduced to the size of 8×8 after excluding the part of the Value. Thus, the dimension of the CT feature is 640 (8×8×5×2=640). Figure 4 shows three target models. For the CT feature, Figure 4b,c is the same and are different from Figure 4a, which can not be distinguished using the color alone. The CT feature has the rotation invariant identity and can distinguish between different texture patterns.

**Figure 5:**
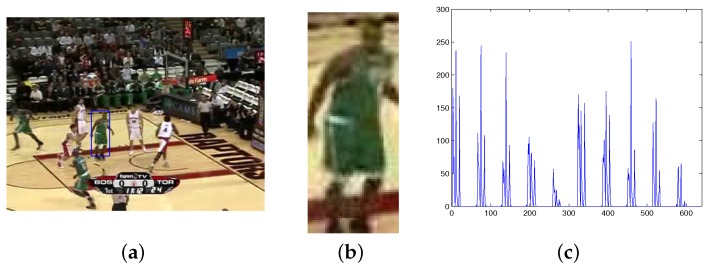
The representation model of the target by the proposed algorithm. (**a**) 1st frame; (**b**) target model region; (**c**) the histogram of CT feature.

The calculation process of the CT feature is as follows. Firstly, let $P_{i}$ be the set of pixels of the target. Calculate $DCS-LBP_{P,R}^{upper}$, $DCS-LBP_{P,R}^{lower}$ and the HSV color space of each point in $P_{i}$ in turn. If the value of $DCS-LBP_{P,R}^{upper}$ or $DCS-LBP_{P,R}^{lower}$ does not belong to the SDCS-LBP, the point will be seen as a meaningless point, which should be eliminated. Secondly, calculate $CT_{P_{i}}^{upper}$ and $CT_{P_{i}}^{lower}$ by multiplying the SDCS-LBP, the Hue and the Saturation. Third, after all the points of the target have been calculated, $his^{upper}\left(H,S,T\right)$ and $his^{lower}\left(H,S,T\right)$ of the target are worked out by putting the CT feature into the histograms. The histogram of the target model (his(CT)) is obtained by combining $his^{upper}\left(H,S,T\right)$ and $his^{lower}\left(H,S,T\right)$. Figure 5 shows the representation of a target model by the proposed method. Figure 5a is the first frame of a sequence. The target is showed in Figure 5b. The histogram of the CT feature is showed in Figure 5c.

## 3. Tracking Algorithm Using the CT Feature

Recently, many similarity measures are used in object tracking algorithms, such as the Euclidean distance, the Bhattacharyya coefficient, the histogram intersection distance, and so on. However, there is still lots of mismatching or misidentification in the tracking process. One of the reasons is that the target model contains some background pixels [15]. This paper proposes using the similarity measure based on maximum posterior probability to solve the problem.

### 3.1. Maximum Posterior Probability Measure

By introducing the candidate area, the maximum posterior probability measure (PPM) is able to decrease the influence of background and increase the importance of the target model in the tracking process. The PPM is a function to evaluate the similarity of the candidate and the target defined as:

(8) $$\rho \left(p,q\right)=\frac{1}{m}\sum_{u=1}^{m_{u}}\frac{p_{u}q_{u}}{s_{u}},$$

where $p_{u}$ and $q_{u}$ are, respectively, the histogram features of the target candidate region and the target model; $s_{u}$ is the feature of the search region of the target candidate; *m* is the pixel number of the target model with $u=1,\cdots ,m_{u}$; and $m_{u}$ is the dimension of feature.

Now, we define a vector ω, which is computed according to Equation (9). u(j) is the feature of the *j*th pixel; $\omega_{j}$ is the PPM of the *j*th pixel of the search region; $A_{i}$ is the set of pixel of the *i*th target candidate region in the search region. Thus, the original PPM can be converted into a simple one as [15]:

(9) $$\begin{array}{c} \rho \left(p^{i},q\right)=\frac{1}{m}\sum_{j\in A_{i}}\omega_{j},\\ \omega_{j}=\left\{\begin{array}{cc} \frac{q_{u(j)}}{s_{u(j)}}, & s_{u(j)}>0,\\ 0, & s_{u(j)}=0. \end{array}\right. \end{array}$$

From the function, it can be found that the PPM and $\omega_{j}$ have a liner relationship. Therefore, we compute the incremental part to obtain the PPM of neighborhood, which makes the recursive algorithm a suitable one.

According to Equation (9), the PPM value of each pixel will be calculated, respectively. Thus, the matching process is simplified to find a target candidate region with the biggest sum of PPM value. The similarity measure of the target candidate and the target model is:

(10) $$\begin{array}{c} \rho_{y_{i}}=\sum_{x_{i}\in A_{y_{i}}}g\left(x_{i}\right),\\ g\left(x_{i}\right)=\frac{q_{u}\left(x_{i}\right)}{s_{u}\left(x_{i}\right)}, \end{array}$$

where ${\left\{x_{i}\right\}}_{i-1,\cdots ,m}$ is the set of pixel’s position with the present frame centered at $y_{i}$; $g(x_{i})$ is the PPM value at $x_{i}$; and $A_{y_{i}}$ is the target candidate centered at $y_{i}$. Supposing the PPM value of each pixel as density and the similarity of the target candidate region as mass, the center of mass $y_{i+1}$ is the target:

(11) $$y_{i+1}=\frac{\sum_{x_{i}\in A_{y_{i}}}x_{i}g\left(x_{i}\right)}{\sum_{x_{i}\in A_{y_{i}}}g\left(x_{i}\right)}.$$

**Figure 6:**
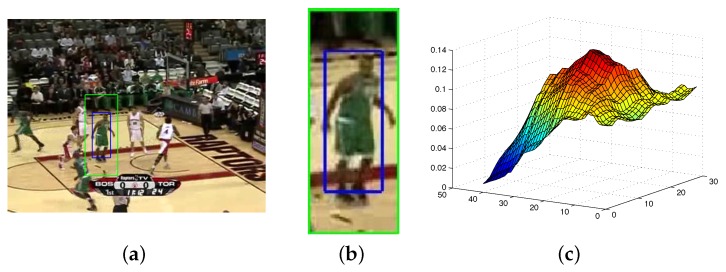
The maximum posterior probability of the target model. (**a**) 1st frame; (**b**) target candidate region; (**c**) the PPM of target model.

Figure 6 shows the PPM of the target model. The target bounded by the blue box and the target candidate region bounded by the green box in Figure 6a are resized. The target model and the target candidate region are showed in Figure 6b. The PPM of the target model, which holds monotonic and distinct peak shapes, is showed in Figure 6c.

### 3.2. Scale Adaptation and Target Model Update

During the tracking process, the target always changes in shape, size, or color. Thus, the target model must be updated. The update must abide by certain rules to prevent the tracking drift. Three strategies are proposed for the target model update.

1. Introduce an adaptive process to fit the target region to a variable target scale for the purpose of precise target tracking.
2. Compute the similarity measure of the scale adapted target. If it is greater than a parameter, update the target model.
3. Introduce a parameter into the tracking algorithm to update part of the target model.

Strategy 1 introduces a scale adaptation function given by [15]:

(12) $$\begin{array}{c} \omega \left(k+1\right)=\left\{\begin{array}{cc} \omega (k+2)+2, & \mathrm{if}\;{\bar{\phi }}_{-1}>0.8\;\mathrm{and}\;{\bar{\phi }}_{0}>0.75\;\mathrm{and}\;{\bar{\phi }}_{1}>0.6,\\ \omega (k-2)-2, & \mathrm{if}\;{\bar{\phi }}_{0}<0.6\;\mathrm{and}\;{\bar{\phi }}_{1}<0.3,\\ \omega (k), & otherwise, \end{array}\right. \end{array}$$

where ω(k) is the size of the target region at frame *k*. $\bar{\phi_{i}}\left(i=-a,\ldots ,0,\ldots ,a\right)$ is the average of the PPM of each pixel. Furthermore, i<0 means the ith outer layer. i=0 represents the target region border. *a* is the comparison step of scale adaptation and is set to 1 without losing the generality. In Equation (12), the expanding condition means the pixels around the border are likely to be a part of the target. The contracting condition means the target region should be reduced consequently. The function is an empirical one. The parameters should be trained by a great number of experiments.

Strategy 2 shows that the frame will not be updated until the similarity measure is greater than a certain parameter. In real scenes, some sudden changes may cause the tracking drift, so the update can not work every frame. *p* is the current frame model, while *q* is the target model. ϕ(p,q) is the similarity of the PPM for the current frame and the target model. If Equation (13) is satisfied, we considered *p* as the reliable CT feature model, and update the target model with *p*:

(13) $$\bar{\phi }\left(p,q\right)\geq \delta .$$

Strategy 3 introduces a parameter into the algorithm to prevent the target model from being updated completely. Because of the limitations of the description to the target model, *p* can not take the place of *q*. The γ parameter is used to partially update the target model:

(14) $$q^{\prime }=\gamma p+(1-\gamma )q,$$

where γ is the update factor; and $q^{\prime }$ is the updated CT feature model. In our experiment, γ is set to be a small value to adapt the changes of the target slowly.

### 3.3. Tracking Algorithm

Initialization: select the target object and compute the histogram his(C,T) of the target model as $q_{u}$. The center of the target $y_{i}$ is the initial position of the tracking object. Let ${\left\{x_{i}\right\}}_{i-1,\cdots ,m}$ be the set of pixel’s position with the present frame centered at $y_{i}$.

1. Set $y_{i}$ as the initial position. Calculate his(C,T) of the search region as $S_{u}$.
2. Calculate the PPM values $g(x_{i})$ of each pixel of the region by Equation (10).
3. Initialize the number of iterations as k=0.
4. Calculate the target location by Equation (11). k=k+1.
5. Repeat Step 4 until $∥y_{i+1}-y_{i}∥<\varepsilon$ or k≥N.
6. Adjust the scale of the target region by Equation (12)
7. Decide whether to update the target by Equation (13). If satisfied, update the target model by Equation (14).
8. Read the next frame of the sequence and turn to Step 1.

If the distance between two iterations is less than ε or the number of iterations exceeds *N*, the algorithm is considered converged.

## 4. Experiments

**Figure 7:**
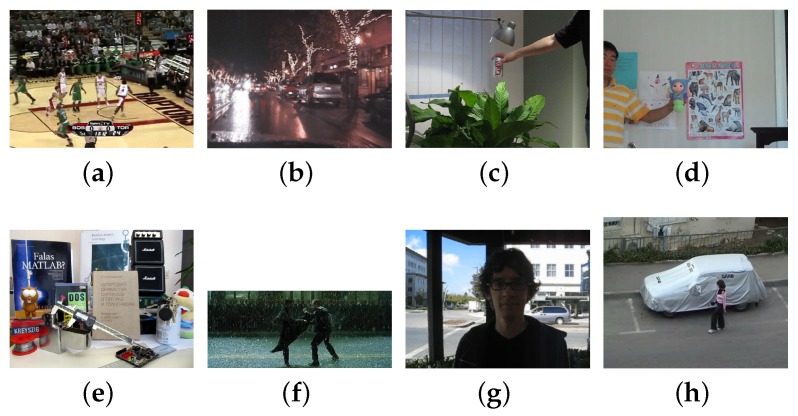
Eight test sequences used in current evaluation. (**a**) basketball; (**b**) car; (**c**) coke; (**d**) doll; (**e**) lemming; (**f**) matrix; (**g**) trellis; (**h**) woman.

The environments are set in some real scenes with similarly colored backgrounds, object occlusions, low illumination color image, and sudden illumination changes [12]. Eight public test sequences are used in experiments which are from the Visual Object Tracking challenge (http://votchallenge.net/index.html) and the Visual Tracker Benchmark [30] (http://www.visual-tracking.net) (see Figure 7). As the visual tracking benchmark, the test sequences are tagged with the following four attributes: low illumination color image (LI), sudden illumination changes (IC), object occlusion (OC), similarly colored background (SCB) (see Table 2). We designed a tracking system based on Matlab R2014a (8.3.0.532). All the trackers run on a standard PC (Intel (R) Core (TM) i5 2.6 GHz CPU with 8 GB RAM).

We compared our algorithm with some state-of-the-art methods including classical mean-shift tracking (KBT) [10], PPM-based color tracking algorithm (PPM) [15], a mean-shift algorithm using the joint color-texture histogram (LBPT) [20] and high-speed tracking with kernelized correlation filters (KCF) [9]. In addition, extra experiments are designed to test the function of the two major parts of the proposed method-the CT feature and the PPM separately. One of the experiments that we use is the CT feature with the Euclidean distance (CT&ED) instead of the PPM as the similarity measure. The other one that we use is the LBP feature with the PPM (LBP&PPM) instead of the CT feature. Both of the two trackers are tested in the experimental framework. All the methods aim at tracking one object in our experiments. The target will be tracked continuously at the rest of the frames.

### 4.1. Parameter Setting

The size of the search region of our methods is set to 2.5 times the target size. In addition, there are five parameters in our tracking algorithm. We set δ=0.85 and γ=0.1 for the target model update in Section 3.2. δ is the control parameter used to determine whether update the model or not. *N* and ε are the iteration parameters for the tracking algorithm in Section 3.3. N=20 is the maximum number of the iteration, and ε=0.5 is the minimum threshold of the iteration. The threshold parameter *T* is important in our algorithm. In order to test the sensitivity of the parameter, the central location error (CLE) is used to describe the tracking result. The CLE is defined as the Euclidean distance between the center of the box predicted by the tracker and that of the box of the ground truth. We set T=1,3,5,7,9 for the calculation of the DCS-LBP. The results of eight test sequences are showed in Table 3. It can be seen that our algorithm performs well on all the tests when T is a small value between 1 to 5. In addition, it only missed the target in the basketball test sequence when T gets larger. Therefore, we set T=1 in the experiments.

### 4.2. Qualitative Comparison

**Figure 8:**
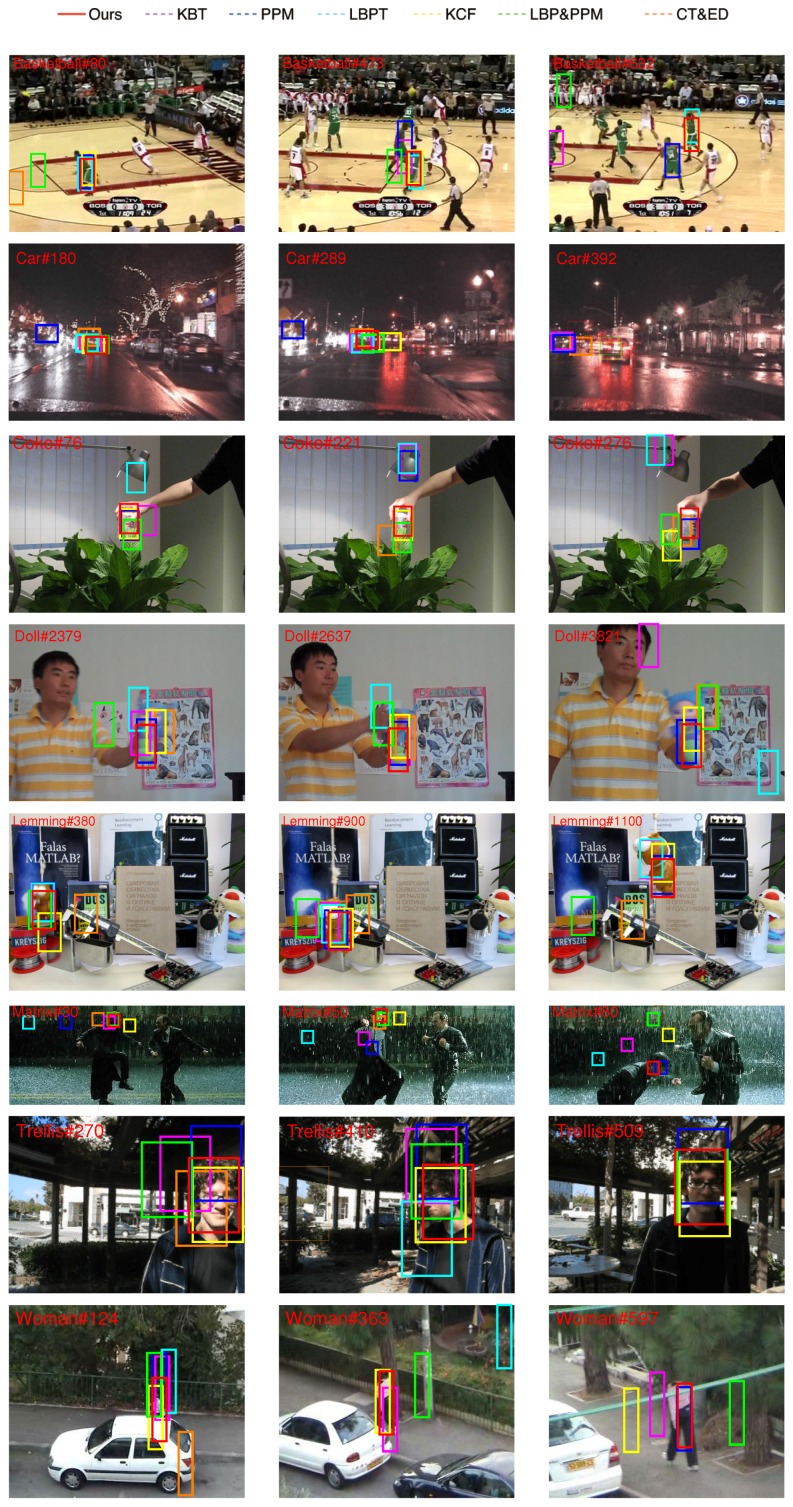
Experiment results of our proposed algorithm, KBT [10], PPM [15], LBPT [20], KCF [9], LBP&PPM and CT&ED on eight challenging sequences (from top to bottom are Basketball, Car, Coke, Doll, Lemming, Matrix, Trellis, Woman, respectively).

Some key frames of each sequence are given in Figure 8. The results of different trackers are shown by the bounding boxes in different colors.

(1) In the basketball sequence, the tracked player moves fast. The environment changes many times. CT&ED lose the target at frame 80. KBT, PPM, and LBP&PPM fail at frame 473, when the player goes through his partner. KCF, LBPT and our tracker can successfully locate the object.
(2) In the car sequence, the target is a car, but the road environment is dark. There are bright lights in the background. All of the trackers can merely track the car in the first 200 frames. However, at frame 260, the car turns right, and only KCF can track the car accurately.
(3) In the coke sequence, the target is a coke and the light changes three times. The coke moves fast and is blocked by plants sometimes. When the coke is blocked by the plants the first time, LBTP misses the target. At frame 221, the occlusion and the illumination happen at the same time, and KBT and PPM obtain the wrong place. During the tracking, both KCF and our method perform better than the others.
(4) The doll sequence has 3872 frames, which is a very long sequence. The target is a doll. It is blocked by the hand, and the scale of it changes sometimes. Because of the similar color with the background, LBP&PPM, LBPT, and CT&ED fail at frame 2378. KCF gives the best result followed by PPM and our tracker.
(5) The lemming sequence is a challenging situation with fast motion, significant deformation and long-term occlusion. KCF missed the target at frame 380 because the target moves fast with the similar background. Our method is more effective than the others during the tracking.
(6) In the matrix sequence, the target is the head. The sequence contains low illumination color image, sudden illumination changes, object occlusion, and similarly colored background. Our tracker gives the best result. At frame 30, all of the methods except ours lose the target. Our tracker misses the target at frame 90, when the target has dramatic changes in shape.
(7) In the trellis sequence, the target is a boy’s face in an outdoor environment. The situation has severe illumination and poses changes. All trackers except KCF and our tracker show some drifting effects at frame 270. The CT&ED loses the target at frame 410. Only KCF and our tracker show a good performance along the whole sequence.
(8) In the woman sequence, the track is a walking woman in the street. The difficulty lies in the fact that the woman is greatly occluded by the parked cars. All the tracks fail at frame 124 except KCF and our tracker because of the occlusion and the small size of the target.

### 4.3. Quantitative Comparison

**Figure 9:**
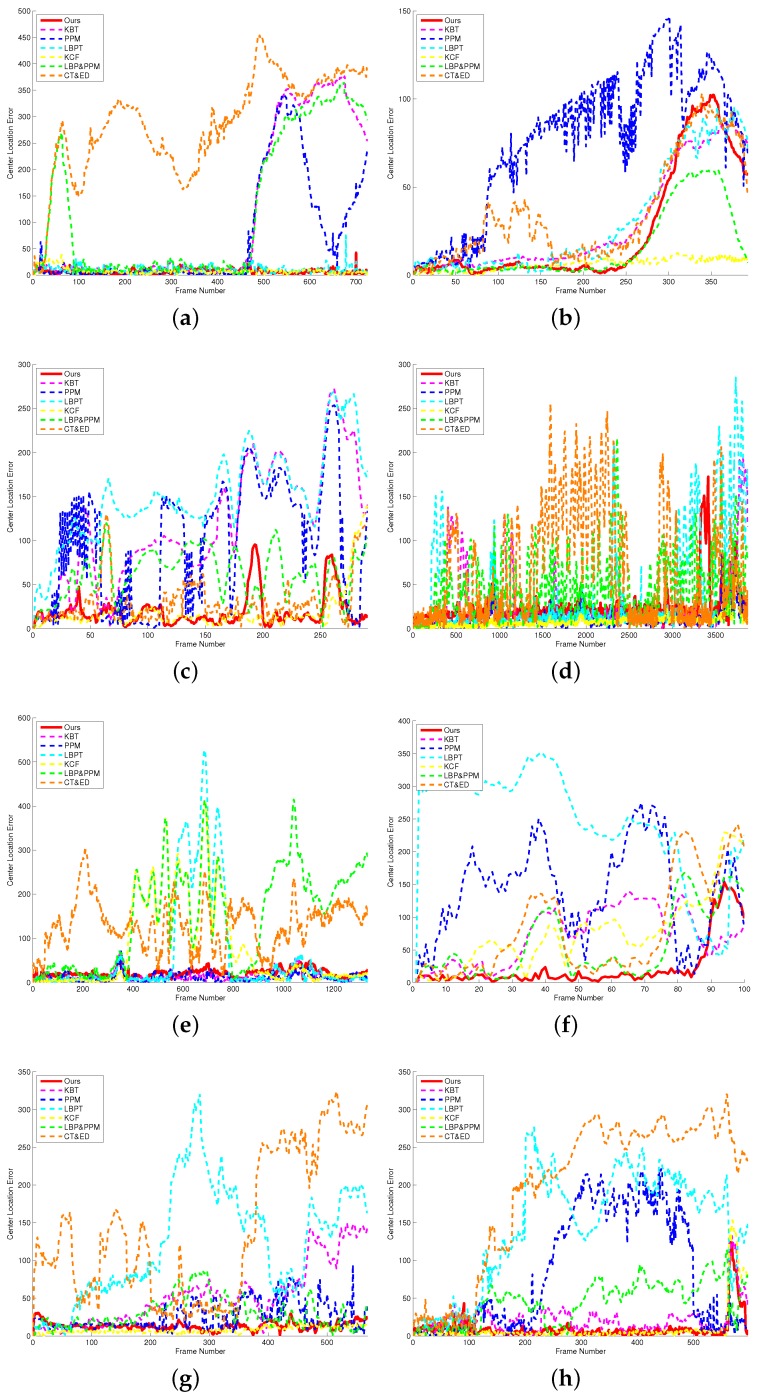
Fame-by-frame comparison of center location errors (in pixels) on eight challenging sequences. Based on the experimental results, our algorithm is able to track targets accurately and stably. (**a**) Basketball; (**b**) Car; (**c**) Coke; (**d**) Doll; (**e**) Lemming; (**f**) Matrix; (**g**) Trellis; (**h**) Woman.

For performance evaluation and comparison, two metrics are considered: the CLE and the success rate (SR), which have been widely used in object tracking [12,31]. A target is considered as successfully tracked if the overlap region between the predicted bounding box and the ground truth exceeds 50% in a frame [32]. The SR is defined as

(15) $$SR=\frac{area(M_{t}\cap M_{g})}{area(M_{t}\cup M_{g})},$$

where $M_{t}$ is the bounding box predicted by the tracker. $M_{g}$ is the ground truth bounding box. The function area(•) means to calculate the area of a region. The CLE has been described in Section 4.1. The results of different methods on eight test sequences are showed in Table 4 and Table 5. It can be seen from Table 4 and Table 5 that our algorithm achieves an SR of 94% and a CLE of 18 which are better than the other algorithms. We also report the central-pixel errors frame-by-frame for each video sequence in Figure 9.

Now, we discuss the influence of the two major parts in our method: the CT feature and the PPM, separately. First, to test the influence of the similarity measure, we compare the trackers using the CT feature and different measures: the Euclidean distance (CT&ED) and the PPM (which is the proposed method—CT&PPM). It can be seen from Table 4 and Table 5 that the PPM achieves an SR of 94% and a CLE of 18, which are better than those achieved by the Euclidean distance (40% and 122%). Second, to test the influence of the feature, we compare the trackers using the PPM and different features: the color feature (PPM), the LBP (LBP PPM) and the CT feature (which is the proposed method—CT&PPM). It can be seen from Table 4 and Table 5 that the CT feature outperforms the others with the highest SR and a lowest CLE. The results demonstrate the effectiveness of both the CT feature and the PPM in improving the tracking accuracy.

### 4.4. Speed Analysis and Discussions

Table 6 lists the needed computation times of the five trackers on our test platform. The trackers run from 160 fps to 60 fps in the current Matlab implementation. The speed of the trackers depends on the area of the candidate region for all the test sequences and the number of iterations. Comparing with KBT, PPM, and KCF, LBPT and the proposed method spend lots of time on texture feature computation. However, they just calculate parts of useful points. Comparing with KBT, KCF and LBPT, PPM and our algorithm can calculate the target model and the search region by joint points to decrease the computational complexity. Because the dimension of the CT feature is 640 compared with KBT, PPM, LBPT, KCF, our tracker takes more time than the other trackers. However, the computational time can satisfy real-time applications.

## 5. Conclusions

A new object tracking method has been proposed in this paper. The algorithm can overcome some difficulties in real scenes such as object occlusion, sudden illumination changes, similarly colored backgrounds, and low illumination color images. This work integrates the outcomes of the color texture feature and PPM centroid iteration tracking. A color texture model called the CT feature is introduced. In addition, we propose using a posterior probability measure with the CT feature for target location. Three target model update strategies are designed to improve the tracking accuracy.

The tracking algorithm only using color can not track the target at similarly colored regions or low illumination regions. The combination of the color and the texture feature can overcome these difficulties, and the SDCS-LBP is a texture feature, which is robust against gray-scale changes. In real scenes, our algorithm shows a good performance. As our method is based on the histograms of the regions, it can overcome the problem of object partial occlusion. PPM measure and the target update strategies can reduce the tracking mistakes. In the experiments, our algorithm performs better than others for most of the test sequences. Future work will be dedicated to decreasing the complexity of the algorithm.

## Figures and Tables

**Table 1:** Examples of five coding rules (T=5).

Image local region	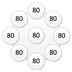	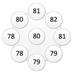	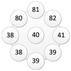
	Texture flat areas	Texture flat areas with noise	Texture change areas
LBP pattern	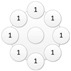	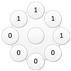	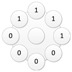
	${\left[11111111\right]}_{2}$	${\left[10000111\right]}_{2}$	${\left[10000111\right]}_{2}$
CS-LBP pattern	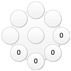	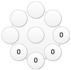	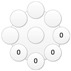
	${\left[0000\right]}_{2}$	${\left[0000\right]}_{2}$	${\left[0000\right]}_{2}$
LTP pattern	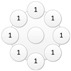	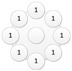	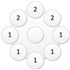
	${\left[11111111\right]}_{3}$	${\left[11111111\right]}_{3}$	${\left[21111122\right]}_{3}$
CS-LTP pattern	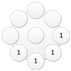	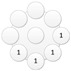	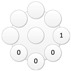
	${\left[1111\right]}_{3}$	${\left[1111\right]}_{3}$	${\left[0001\right]}_{3}$
DCS-LTP pattern	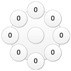	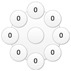	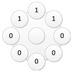
	${\left[0000\right]}_{2}{\left[0000\right]}_{2}$	${\left[0000\right]}_{2}{\left[0000\right]}_{2}$	${\left[1000\right]}_{2}{\left[0011\right]}_{2}$

**Table 2:** Eight sequences in the experiment.

Sequences	Size	Frame	fps	Object Number	Attributes
car	320 × 240	368	30	1	IC SCB LI
basketball	576 × 432	725	30	>8	IC OC SCB
coke	640 × 480	291	30	1	IC OC SCB
doll	400 × 300	3872	30	1	IC OC
lemming	640 × 480	1336	30	1	IC OC
matrix	800 × 336	100	30	2	IC OC SCB LI
Trellis	320 × 240	569	30	1	IC SCB LI
woman	352 × 288	597	30	1	IC OC

**Table 3:** The parameter setting (CLE).

SEQUENCE	T = 1	T = 3	T = 5	T = 7	T = 9
basketball	7	21	20	278	255
car	25	27	27	27	25
coke	19	18	17	14	16
doll	26	27	23	25	26
lemming	21	20	20	21	22
matrix	23	24	24	24	24
Trellis	13	13	12	12	12
woman	10	7	9	11	8
Average CLE	18	20	19	52	49

**Table 4:** Success rates (%) of the proposed method compared with the other trackers.

SEQUENCE	KBT [10]	PPM [15]	LBPT [20]	KCF [9]	Proposed	LBP&PPM	CT&ED
basketball	65	68	100	100	100	56	3
car	65	20	63	100	71	76	51
coke	18	37	7	94	94	48	89
doll	88	100	79	100	97	57	56
lemming	99	99	83	68	100	38	24
matrix	41	15	7	31	91	57	49
Trellis	67	90	27	100	100	87	27
woman	93	53	19	94	95	42	18
Average success rate	67	60	48	86	94	58	40

**Table 5:** Center location errors of the proposed method compared with the other trackers (pixels).

SEQUENCE	KBT [10]	PPM [15]	LBPT [20]	KCF [9]	Proposed	LBP&PPM	CT&ED
basketball	113	68	11	8	7	123	288
car	29	77	31	6	25	16	36
coke	119	99	153	19	19	64	31
doll	25	12	42	8	26	51	67
lemming	13	12	61	78	20	149	132
matrix	75	14	249	76	23	61	85
Trellis	54	26	123	8	13	30	142
woman	22	85	145	10	10	46	196
Center location error	56	49	102	27	18	66	122

**Table 6:** Computation speed comparison (fps).

SEQUENCE	KBT [10]	PPM [15]	LBPT [20]	KCF [9]	Proposed
Average success rate	164	100	88	165	66

## References

[1] Welch G., Bishop G. (1997). SCAAT: Incremental Tracking with Incomplete Information. Proceedings of the 24th Annual Conference on Computer Graphics and Interactive Techniques.

[2] Isard M., Blake A. (1998). Condensation—Conditional Density Propagation for Visual Tracking. Int. J. Comput. Vis..

[3] Choo K., Fleet D. People tracking using hybrid Monte Carlo filtering. Proceedings of the Eighth IEEE International Conference on Computer Vision.

[4] Lucas B.D., Kanade T. An iterative image registration technique with an application to stereo vision. Proceedings of the IJCAI’81 Proceedings of the 7th international joint conference on Artificial intelligence.

[5] Reid D. (1979). An algorithm for tracking multiple targets. IEEE Trans. Autom. Control.

[6] Cox I., Hingorani S. (1996). An efficient implementation of Reid’s multiple hypothesis tracking algorithm and its evaluation for the purpose of visual tracking. IEEE Trans. Pattern Anal. Mach. Intell..

[7] Comaniciu D., Ramesh V., Meer P. (2003). Kernel-based object tracking. IEEE Trans. Pattern Anal. Mach. Intell..

[8] Jepson A., Fleet D., El-Maraghi T. (2003). Robust online appearance models for visual tracking. IEEE Trans. Pattern Anal. Mach. Intell..

[9] Henriques J.F., Caseiro R., Martins P., Batista J. (2015). High-Speed Tracking with Kernelized Correlation Filters. IEEE Trans. Pattern Anal. Mach. Intell..

[10] Comaniciu D., Ramesh V., Meer P. Real-time tracking of non-rigid objects using mean shift. Proceedings of the Computer Vision and Pattern Recognition.

[11] Cai Y., Freitas N.D., Little J.J., Leonardis A., Bischof H., Pinz A. (2006). Robust Visual Tracking for Multiple Targets. Computer Vision–ECCV 2006.

[12] Kim D., Kim H., Lee S., Park W., Ko S. (2014). Kernel-Based Structural Binary Pattern Tracking. IEEE Trans. Circ. Syst. Video Technol..

[13] Joukhadar A., Scheuer A., Laugier C. Fast contact detection between moving deformable polyhedra. Proceedings of the 1999 IEEE/RSJ International Conference on Intelligent Robots and Systems.

[14] Liu T.L., Chen H.T. (2004). Real-time tracking using trust-region methods. IEEE Trans. Pattern Anal. Mach. Intell..

[15] Feng Z., Lu N., Jiang P. (2008). Posterior probability measure for image matching. Pattern Recognit..

[16] Haritaoglu I., Flickner M. Detection and tracking of shopping groups in stores. Proceedings of the 2001 IEEE Computer Society Conference on Computer Vision and Pattern Recognition.

[17] Heikkila M., Pietikainen M. (2006). A texture-based method for modeling the background and detecting moving objects. IEEE Trans. Pattern Anal. Mach. Intell..

[18] Collins R.T., Liu Y., Leordeanu M. (2005). Online selection of discriminative tracking features. IEEE Trans. Pattern Anal. Mach. Intell..

[19] Wang J., Yagi Y. (2008). Integrating color and shape-texture features for adaptive real-time object tracking. IEEE Trans. Image Process..

[20] Ning J., Zhang L., Zhang D., Wu C. (2009). Robust object tracking using joint color-texture histogram. Int. J. Pattern Recognit. Artif. Intell..

[21] Lowe D.G. (2004). Distinctive image features from scale-invariant keypoints. Int. J. Comput. Vis..

[22] Ke Y., Sukthankar R. PCA-SIFT: A more distinctive representation for local image descriptors. Proceedings of the 2004 IEEE Computer Society Conference on Computer Vision and Pattern Recognition.

[23] Mikolajczyk K., Schmid C. (2005). A performance evaluation of local descriptors. IEEE Trans. Pattern Anal. Mach. Intell..

[24] Bay H., Tuytelaars T., van Gool L. (2006). Surf: Speeded up robust features. Computer Vision–ECCV 2006.

[25] Alahi A., Ortiz R., Vandergheynst P. Freak: Fast retina keypoint. Proceedings of the 2012 IEEE Conference on Computer Vision and Pattern Recognition (CVPR).

[26] Ojala T., Pietikainen M., Maenpaa T. (2002). Multiresolution gray-scale and rotation invariant texture classification with local binary patterns. IEEE Trans. Pattern Anal. Mach. Intell..

[27] Ahonen T., Hadid A., Pietikainen M. (2006). Face description with local binary patterns: Application to face recognition. IEEE Trans. Pattern Anal. Mach. Intell..

[28] Heikkilä M., Pietikäinen M., Schmid C. (2009). Description of interest regions with local binary patterns. Pattern Recognit..

[29] Tan X., Triggs B. (2010). Enhanced Local Texture Feature Sets for Face Recognition Under Difficult Lighting Conditions. IEEE Trans. Image Process..

[30] Wu Y., Lim J., Yang M.H. Online Object Tracking: A Benchmark. Proceedings of the IEEE Conference on Computer Vision and Pattern Recognition (CVPR).

[31] Wang N., Wang J., Yeung D.Y. Online Robust Non-negative Dictionary Learning for Visual Tracking. Proceedings of the 2013 IEEE International Conference on Computer Vision (ICCV).

[32] Everingham M., Gool L.V., Williams C.K.I., Winn J., Zisserman A. (2010). The Pascal Visual Object Classes (VOC) Challenge. Int. J. Comput. Vis..

